# Qualitative Study of Functional Groups and Antioxidant Properties of Soy-Based Beverages Compared to Cow Milk

**DOI:** 10.3390/antiox4030523

**Published:** 2015-07-15

**Authors:** Alessandra Durazzo, Paolo Gabrielli, Pamela Manzi

**Affiliations:** Consiglio per la ricerca in agricoltura e l’analisi dell’economia agraria—Centro di ricerca per gli alimenti e la nutrizione (CRA-NUT), Via Ardeatina 546, Roma 00178, Italy; E-Mails: paolo.gabrielli@entecra.it (P.G.); pamela.manzi@entecra.it (P.M.)

**Keywords:** soy-based beverages, color, antioxidants, polyphenols, FTIR-ATR, cow milk

## Abstract

Soy-based beverages are a source of high quality proteins and balanced nutrients; they thus represent an alternative to milk in case of allergy to cow milk proteins or intolerance to lactose. In this research, antioxidant properties of soy-based beverages and UHT cow milk were studied. In addition, color parameters, by a quick and non-destructive methodology, were studied in order to verify a possible correlation with antioxidant properties and a qualitative analysis of the major functional groups undertaken by Fourier Transformed Infrared Spectroscopy (FTIR) on Attenuated Total Reflectance (ATR) was carried out. Extractable and hydrolysable polyphenols were studied in soy-based beverages. However, only the extractable fraction was studied in UHT milk, which was characterized by a small amount of polyphenols. All color parameters showed highly significant differences among soy-based beverages and between soy-based beverages and cow milk. FTIR-ATR spectra of soy-based beverages and cow milk showed several differences in the various regions depending on both the specific contribution of molecular groups and different food items.

## 1. Introduction

Soy-based beverages, also called soy milks, soymilks or soybean milks, are the aqueous extracts of whole soybeans (*Glicine max*) and are a source of high quality protein and balanced nutrients. Soy-based beverages are a valid alternative to cow milk: milk and dairy products are considered a well-balanced food as they are a source of essential amino acids, vitamins and calcium; however, their consumption must be avoided in case of allergy to cow milk proteins or severe intolerance to lactose. The consumer acceptability of soy-based beverages might be affected by their astringent flavor [1,2], nevertheless there is an increased interest among consumers. New formulations of soy-based beverages with several ingredients have been launched on the market and nowadays new technologies have allowed the improvement of their sensory characteristics, and their consumption is increasingly spreading worldwide [3], especially among vegetarians. Soy-based products have a wide applicability in the food industry: they are used directly as a beverage or as an ingredient in yogurt, infant formulas and/or desserts.

Soy-based beverages are free of cholesterol and lactose and, moreover, have small amounts of saturated fatty acids and different phytochemicals with beneficial properties, *i.e.*, isoflavones or tocopherols that contribute to their antioxidant properties [4,5]. The concentrations of these bioactive compounds and their antioxidant properties are affected by food matrix and processing condition techniques, such as heat treatment, dilution with non-soy ingredients, enzyme hydrolysis and fermentation [6,7,8,9,10]. Recently, different studies have shown a close relationship between consumption of soy-based foods and potential health benefits related to cardiovascular diseases, menopausal symptoms, osteoporosis, breast and prostate cancers [11,12].

The investigation of antioxidant properties could represent a first stage of evaluation of the potential beneficial properties of foods.

The chemical diversity of each compound, their interactions and their different mechanisms of action makes difficult an appropriate and right determination of the antioxidant activity.

Extraction, antioxidant capacity measurements and expression of results represents the fundamental steps in the evaluation of antioxidant properties [13,14].

Chemical extraction is affected by several factors such as type of solvents, extraction time and temperature as well as by the chemical composition and physical characteristics of the sample analyzed [15].

Various procedures for reaching an optimal antioxidant extraction have been developed over the years: ethanol, methanol and acetone and/or their mixtures were used as extraction solvents; in particular, acid methanol/water mixture was utilized to improve extraction. Recent studies have performed an alkaline hydrolysis, acid hydrolysis or enzymatic digestion.

Nowadays, the distinction between extractable and non extractable antioxidants represents an important element for defining the beneficial properties of food matrixes.

Literature data reported that the use of at least two or three assays is strongly recommended for assessing antioxidant properties [16].

This research is a preliminary study on characterization of soy-based beverages, purchased in supermarkets and then, as additional information, UHT cow milk was used as a comparison. This work particularly focused on the development of an optimal extraction procedure of antioxidants and the evaluation of antioxidant properties in these food items. In addition, color parameters, by a quick and non-destructive methodology, were studied in order to verify a possible correlation with antioxidant properties and a qualitative analysis of the major functional groups undertaken by Fourier Transformed Infrared Spectroscopy (FTIR) on Attenuated Total Reflectance (ATR) was carried out.

## 2. Experimental Section

### 2.1. Sampling

UHT cow milk and commercially available brands of soy-based beverages were purchased from local supermarkets. Three different batches of UHT milk and soy-based beverages were analyzed.

UHT milk was chosen as a comparison because it is the most commonly consumed in Italy and moreover it is liquid, not reconstituted and is stored at room temperature like the selected soy-based beverages.

The ingredient list of soy-based beverages and UHT milk was reported as follows:

S1: Tonyu (water, dehulled soybean), sugar cane, calcium carbonate, natural vanilla flavor, salt, stabilizers: microcrystalline cellulose, guar seed flour; cholecalciferol, corn maltodextrin.
S2 Water, soy beans shelled, sugar, tricalcium phosphate, acidity regulator, salt, flavor, stabilizer, vitamins.
S3: Water, soybeans, cane sugar, Alga Lithothamnium calcareum, vanilla.
UHT: Cow milk

### 2.2. Chemicals

Reagents and standards were obtained from Sigma Chemical Co. (St. Louis, MO, USA). Ultrapure water was obtained by an ion exchange system to >18 mΩ resistivity (Millipore, Billerica, MA, USA).

### 2.3. Color Measurements

Color measurement was performed by a handheld tristimulus colorimeter (Konica Minolta CR-400, Chiyoda-ku, Tokyo, Japan), using Commission Internationale de l’Eclairage (CIE) standard illuminant D65, observation angle of 0° and 8 mm diameter of field of view. A white calibration plate was used to calibrate. Each sample was examined and averaged to determine L*, a* and b* values.

### 2.4. Total Polyphenol Content (TPC) and Antioxidant Properties

Total polyphenols were extracted as described by Durazzo *et al.* [17] with modifications. Aqueous-organic extracts (extractable polyphenols) and their residues (hydrolysable polyphenols) were isolated and studied for soy-based beverages, while aqueous-organic extracts (extractable polyphenols) were studied only in UHT milk because of the nature of this food matrix and its related composition in bioactive components.

Aqueous-organic extract. About 5.5–6.0 g of soy-based beverages or 9.0–10.0 g of cow milk were placed in a test tube, and 20 mL of acid methanol/water (50:50 v/v, pH 2) were added. Tubes were swirled at room temperature for 3 min, gently shaken for 1 h in a water bath at room temperature as well, and then centrifuged at 2500× *g* for 10 min. The supernatant was recovered. Then, an extraction with 20 mL acetone/water (70:30, v/v) was carried out as reported above. Methanolic and acetonic extracts were combined and centrifuged at 2800× *g* for 15 min for better cleanliness. The resulting mixture was used for the assays as follows.

Residue. 500 mg of soy-based beverage residue, dried by N_2_ flow, was mixed with 20 mL of methanol and 2 mL of sulphuric acid (18 M). Samples were gently stirred for 1 min and shaken at 85 °C for 20 h in a water bath, centrifuged (2500× *g* for 10 min) and the supernatant was recovered. After two washings with ultrapure water, the final volume was taken up to 50 mL. The tube was centrifuged at 2800× *g* for 20 min and the resulting supernatant was used for the assays as follows.

Total Polyphenol Content (TPC). This assay was carried out using the Folin-Ciocalteau procedure [18]*.*

Antioxidant properties. This determination was carried out by the FRAP (Ferric Reducing Antioxidant Power) assay according to the methods of Benzie and Strain [19] and Pulido *et al.* [20].

### 2.5. FTIR-ATR Spectra

The FTIR-ATR spectra (Nicolet iS10 FT-IR spectrometer, Thermo Fisher Scientific, Waltham, MA, USA) equipped with a diamond crystal cell (ATR) were acquired at 8 cm^−1^ resolution, 50 scans of each sample [21], CO_2_ atmospheric correction, in the wavenumber range of 4000–600 cm^−1^.

A background spectrum was scanned by filling the diamond crystal cell ATR with ultrapure water in order to correct the sample spectra. After scanning each sample, the ATR crystal was cleaned with propan-2-ol and ethanol: the cleanliness of ATR crystal was verified. Measurements were repeated 10 times and spectra were averaged.

### 2.6. Statistical Analysis

Data are shown as mean value with standard deviation. Statistica for Windows (Statistical package; release 4.5; StatSoft Inc., Tulsa, OK, USA) was used to perform One-way Analysis of Variance (ANOVA). *p*-Values < 0.05 were considered significant.

## 3. Results and Discussion

### 3.1. Color Parameters

**Figure 1:**
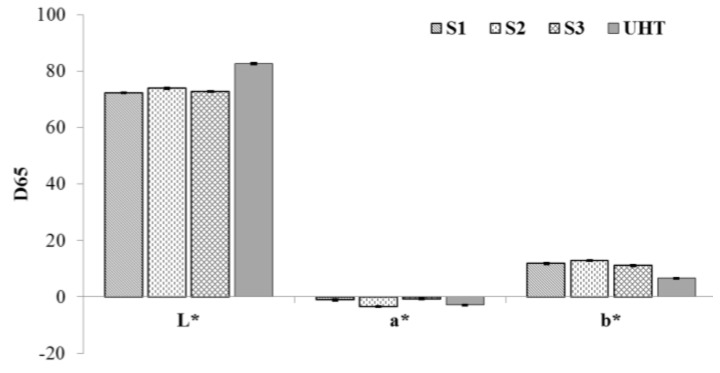
Color parameters of soy-based beverages and UHT cow milk.

Color, a rapid measurement, is important because it is the first quality parameter evaluated by consumers [22] and it was often related to antioxidant properties or an indicator for severe heat treatments [23]. In Figure 1 color parameters (L*, a* and b*) of soy-based beverages and UHT cow milk were reported. Brightness (L*), redness (a*) and yellowness (b*) showed highly significant (*p* < 0.0001) differences among soy-based beverages and between soy-based beverages and cow milk. Soy-based beverages showed L* values ranging from 72.25 to 73.91, lower than those obtained for UHT milks (L* 82.63). Soy-based beverages showed a marked yellow component (11.15 < b* < 12.86) in relation to cow milk (b* 6.55), and a* values were significantly different (*p* < 0.0001) among soy-based beverages (−3.38 < a* < −0.78) and between UHT milk (a* −2.85) and each soy-based beverage.

The color parameters in milk were also related to storage, temperature of processing and chemical reactions such as Maillard’s reaction [24]. In a previous work [25] the b* values of whole milk, that increased with the temperature of treatment (pasteurized milk, microfiltered milk and UHT), were in accordance with milk samples studied in this research.

### 3.2. Antioxidant Properties and Total Polyphenol Content

Antioxidant properties, measured by FRAP assay, were presented in Table 1. Values varied significantly (*p* < 0.05) among products: in soy-based beverages FRAP values ranged from 8.54 to 16.27 µmol/g dry matter in aqueous-organic extracts and between 12.79 to 18.98 µmol/g dry matter in residues. In UHT milk, samples exhibited an average FRAP value of 3.10 µmol/g dry matter (Table 1). Rau De Almeida Callou *et al.* [7] studied different brands of soy-based beverages (including products from soy protein isolate and soy milk, mixed with fruit juice) and their results showed that antioxidant activity varied significantly among products. Moreover, storage at room temperature caused a significant decrease of antioxidant capacity, soluble phenolics and isoflavone contents.

**Table 1:** FRAP and TPC values of soy-based beverages and UHT cow milk.

Samples	FRAP (µmol/g dry matter)		TPC (mg/100g dry matter)	
	Aqueous-Organic Extract	Residue	Aqueous-Organic Extract	Residue
S1	16.27 ± 0.43 ^d^	12.79 ± 1.72 ^a^	225.18 ± 14.19 ^d^	1468.77 ± 57.62 ^a^
S2	13.49 ± 1.50 ^c^	18.98 ± 1.24 ^b^	197.22 ± 10.86 ^c^	2511.16 ± 244.54 ^b^
S3	8.54 ± 0.16 ^b^	13.84 ± 1.50 ^a^	113.38 ± 9.06 ^b^	1317.60 ± 160.10 ^a^
UHT	3.10 ± 0.16 ^a^	n.d.	51.18 ± 2.66 ^a^	n.d.

Data are mean ± standard deviation; by column, values with different superscript letters are significantly different (*p* < 0.05). n.d. = not detected. FRAP, Ferric Reducing Antioxidant Power; TPC, Total Polyphenol Content.

In Table 1, Total Polyphenol Content (TPC) of soy-based beverages and UHT milk was showed. In soy-based beverages the contribution of extractable polyphenols (aqueous-organic extracts) and hydrolysable polyphenols (residue) to antioxidant properties varied, respectively, from 38% to 56% and from 44% to 62%; both extractable and hydrolysable polyphenols are important fractions for antioxidant properties in accordance with data reported in literature [13,26]. Data of TPC in soy-based beverages showed a good correlation with FRAP ones (aqueous-organic extract *r* = 0.99; residue *r* = 0.96). In aqueous-organic extracts, TPC values were in the following order of the concentrations S1 > S2 > S3 whereas in residue, S2 reached the highest values: this behavior is probably due to the formulation of these products. Some authors [9] showed good correlations of both DPPH (1,1-diphenyl-2-picrylhydrazyl) and FRAP assays with total flavonoid and total polyphenol content (*p* < 0.01) by investigating phenols, flavonoids and antioxidant activity of soy-milks from different soybean cultivars.

Moreover, as expected, FRAP values and TPC data of aqueous-organic extracts showed lower values in UHT cow milk than soy-based beverages (Table 1). This is probably due to the profile in bioactive components of milk [25,27], food matrix of animal origin characterized by a small amount of polyphenols compared to those of vegetable origin.

However, severe heat treatments, such as UHT, in milk can be associated with the development of brown melanoidins due to Maillard reaction that could be responsible of the increase of antioxidant properties [28,29]. In further researches, the antioxidant properties of different heat treated cow milk (raw, pasteurizated, high pasteurizated or microfiltered) will be carried out.

Furthermore, the antioxidant properties FRAP value and Total Polyphenol Content of soy-based beverages and UHT milk were related with color parameter: b* value showed a good Pearson correlation with FRAP in aqueous-organic extracts and residues (*r* = 0.88 and 0.84 respectively) and with TPC in aqueous-organic extracts and residues (*r* = 0.87 and 0.96 respectively).

### 3.3. FT-IR ATR Spectra

Fourier Transform Infrared (FTIR) spectroscopy is a rapid technique for the determination of the “fingerprint” of organic compounds because their functional groups exhibit characteristic vibrational absorption frequencies in specific infrared regions.

In all samples, as water was over 83%, spectra were corrected with the spectrum of water according to some authors [21,30] because typical absorption bands for water, located between 3650–3000 cm^−^^1^ and 1680–1600 cm^−1^ [31], are able to obscure the absorption of the studied samples.

**Figure 2:**
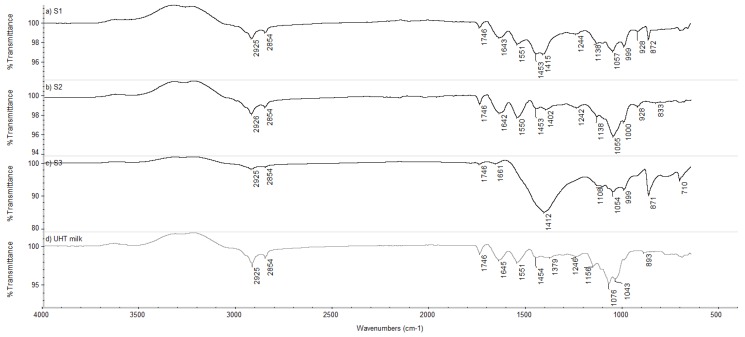
Fourier Transformed Infrared Spectroscopy on Attenuated Total Reflectance (FTIR-ATR) spectra of soy-based beverages (**a**) S1; (**b**) S2; (**c**) S3 and UHT cow milk (**d**) corrected with a background spectrum of water.

FTIR-ATR spectra (Figure 2) were characterized by several typical peaks that could be related to the specific contribution of molecular groups.

The transmission measurements observed at 1651 cm^−1^ are attributed to amide I band due to C=O stretching [32], and at 1545 cm^−1^ are attributed to amide II band due to N−H bending. Amide groups showed higher transmittance measurements in soy-based beverages than UHT cow milk (Figure 2). Several authors showed pure soybean proteins exhibit specific absorption bands in the regions of amide I, II and III [33]. Jaiswal *et al.* [34] showed differences within the wavenumber range of 1680–1058 cm^−1^ when studying milk, soymilk and adulterated milk samples.

The region located at 1200–900 cm^−1^ can be associated to polysaccharide peaks related to C−C and C-O stretching modes; in soymilks this region has higher intensity than UHT cow milk because of the presence of added sugars, as confirmed in the ingredient list. Moreover it is worth highlighting that in cow milk the major contribution to the polysaccharide band (1076 cm^−1^) is due to lactose, absent in soy-milk based beverages.

Regarding the evaluation of specific functional groups, interesting differences were identified in the region of 1680–900 cm^−1^. This fingerprint region includes the absorption frequency of amides, saccharide structures and specific groups that characterize several bioactive molecules (e.g., α-tocopherol, phytates, *etc.*).

## 4. Conclusions

In this preliminary research, some soy-based beverages and UHT cow milk were studied in order to investigate antioxidant properties, color parameters and a qualitative analysis of their major functional groups. All color parameters showed highly significant differences among soy-based beverages and between soy-based beverages and cow milk. Moreover, this study provided a fingerprint of the most representative functional groups of soy-based beverages and highlighted their interesting antioxidant properties. Results confirmed the great variability of soy-based beverages available on the market and highlighted that soy-based beverages, in contrast to cow milk, have several added ingredients such as sugar, flavors or stabilizers.
